# Antisense RNA foci in the motor neurons of *C9ORF72*-ALS patients are associated with TDP-43 proteinopathy

**DOI:** 10.1007/s00401-015-1429-9

**Published:** 2015-05-06

**Authors:** Johnathan Cooper-Knock, Adrian Higginbottom, Matthew J. Stopford, J. Robin Highley, Paul G. Ince, Stephen B. Wharton, Stuart Pickering-Brown, Janine Kirby, Guillaume M. Hautbergue, Pamela J. Shaw

**Affiliations:** Sheffield Institute for Translational Neuroscience (SITraN), University of Sheffield, 385A Glossop Road, Sheffield, S10 2HQ UK; Institute of Brain, Behaviour and Mental Health, 2.014 AV Hill Building, University of Manchester, Manchester, M13 9PT UK

**Keywords:** *C9ORF72*, Amyotrophic lateral sclerosis, RNA foci, Dipeptide repeat protein, Immunohistochemistry

## Abstract

GGGGCC repeat expansions of *C9ORF72* represent the most common genetic variant of amyotrophic lateral sclerosis (ALS) and frontotemporal dementia. We and others have proposed that RNA transcribed from the repeat sequence is toxic via sequestration of RNA-binding factors. Both GGGGCC-repeat (sense) and CCCCGG-repeat (antisense) molecules are detectable by fluorescence in situ hybridisation as RNA foci, but their relative expression pattern within the CNS and contribution to disease has not been determined. Blinded examination of CNS biosamples from ALS patients with a repeat expansion of *C9ORF72* showed that antisense foci are present at a significantly higher frequency in cerebellar Purkinje neurons and motor neurons, whereas sense foci are present at a significantly higher frequency in cerebellar granule neurons. Consistent with this, inclusions containing sense or antisense derived dipeptide repeat proteins were present at significantly higher frequency in cerebellar granule neurons or motor neurons, respectively. Immunohistochemistry and UV-crosslinking studies showed that sense and antisense RNA molecules share similar interactions with SRSF2, hnRNP K, hnRNP A1, ALYREF, and hnRNP H/F. Together these data suggest that, although sense and antisense RNA molecules might be expected to be equally toxic via their shared protein binding partners, distinct patterns of expression in various CNS neuronal populations could lead to relative differences in their contribution to the pathogenesis of neuronal injury. Moreover in motor neurons, which are the primary target of pathology in ALS, the presence of antisense foci (*χ*^2^, *p* < 0.00001) but not sense foci (*χ*^2^, *p* = 0.75) correlated with mislocalisation of TDP-43, which is the hallmark of ALS neurodegeneration. This has implications for translational approaches to *C9ORF72* disease, and furthermore interacting RNA-processing factors and transcriptional activators responsible for antisense versus sense transcription might represent novel therapeutic targets.

**Electronic supplementary material:**

The online version of this article (doi:10.1007/s00401-015-1429-9) contains supplementary material, which is available to authorized users.

## Introduction

GGGGCC hexanucleotide repeat expansions in *C9ORF72* represent the most common genetic variant of amyotrophic lateral sclerosis (ALS) and frontotemporal dementia (FTD) [7, 27]. The mechanism of pathogenesis is unknown, but it has been suggested that a gain-of-function toxicity may be mediated via sequestration of RNA recognition motif (RRM) containing proteins by RNA foci [2, 3]. It has been observed that RNA foci are formed, not only from sense, but also from antisense transcription of the repeat expansion [7, 16, 21]. The relative contribution of GGGGCC-repeat (sense) and CCCCGG-repeat (antisense) RNA molecules to disease pathogenesis is unknown, but is likely to have significant implications for subsequent translational research. Work by Haeusler et al. [11] recently suggested that, with a small number of exceptions, the protein binding partners of the two species of RNA foci are similar.

Another suggested mechanism of pathogenesis is direct toxicity of one or more of five dipeptide repeat proteins (DPRs) translated in different reading frames from either the sense [23] or antisense [24] RNA molecules. Poly(Gly-Ala) (GA) and poly(Gly-Arg) (GR) are translated from sense RNA molecules; poly(Pro-Ala) (PA) and poly(Pro-Arg) (PR) are translated from the antisense RNA molecules and poly(Pro-Gly) (PG) is translated from both molecules. Several recent studies have described how these proteins might disrupt ribosomal RNA biogenesis and pre-mRNA splicing [15, 22] or form toxic aggregates [20]. If DPRs are key to pathogenesis, then aberrant nuclear export of repeat RNA sequences, which is necessary to facilitate access to cytoplasmic translation machinery, may be an attractive therapeutic target. We have previously identified interactions between sense RNA repeat sequences and mRNA export adaptor proteins which might have a role in inappropriate licencing for nuclear export [3].

We conducted extensive immunohistochemistry (IHC) in tissue from *C9ORF72*-ALS cases to determine the distribution of each species of RNA foci within various CNS neuronal populations known to degenerate in *C9ORF72*-disease [19]. Blinded examination of serial sections showed that antisense foci are present at a higher frequency in cerebellar Purkinje neurons and motor neurons, whereas sense foci are present at a higher frequency in cerebellar granule neurons. Similar examination in neuronal populations of the hippocampal dentate gyrus and CA4 subfield did not reveal a consistent distinction, with significant variability between cases. Moreover, neuronal inclusions containing DPRs translated from sense RNA are present at a higher frequency in cerebellar granule neurons, whereas neuronal inclusions containing DPRs translated from antisense RNA are present at a higher frequency in motor neurons. Notably, motor neurons are the primary target of pathology in ALS. Furthermore we examined the distribution of RRM-containing proteins predicted to bind one or both of sense and antisense foci with specific attention to colocalisation with antisense RNA foci. Direct and specific binding to the antisense/sense repeat sequence was examined by UV crosslinking using purified recombinant proteins. Finally, we studied the relative association of each species of RNA foci with the hallmark of ALS neurodegeneration, namely mislocalisation of TDP-43 in motor neurons [25]. We add novel insights to this field—in particular our focus on neuropathology has allowed us to contextualize the sense and antisense RNA foci within framework of the human disease.

## Materials and methods

### Human samples

This study was approved by the South Sheffield Research Ethics Committee and informed consent was obtained for all samples. Brain and spinal cord tissues were donated to the Sheffield Brain Tissue Bank for research, with the consent of the next of kin. IHC and RNA fluorescence in situ hybridisation (FISH) were performed on formalin fixed paraffin-embedded (FFPE) tissues from eight *C9ORF72*+ patients with ALS and/or FTD, three non-*C9ORF72* ALS patients, and three neurologically normal controls. Clinical features of cases examined are summarized in Table 1.

**Table 1:** Clinical details of *C9ORF72*+ cases used in pathological analysis

Case	Phenotype	Sex (M/F)	Age at onset (Years)	Disease duration (Months)	Site of onset	Post-mortem delay (h)
1	ALS-FTD	F	63	43	Cognitive	24
2	ALS	F	56	43	Limb	32
3	ALS	M	69	38	Limb	~96
4	ALS	F	61	40	Bulbar	7
5	ALS	F	58	7	Limb	2
6	ALS	M	62	20	Bulbar	~48
7	ALS	F	50	28	Bulbar	22
8	FTD	F	58	36	Cognitive	N/A

Case numbers are matched in Tables 1, 2, and 3

### RNA FISH

A 5′ TYE-563-labelled LNA (16-mer fluorescent)-incorporated DNA probe was used against the sense (Exiqon, Inc.; batch number 607323) and the antisense RNA hexanucleotide repeat (Exiqon, Inc.; batch number 610331). Slides were prepared and RNA foci were visualised as described previously [3] using a Leica SP5 confocal microscope system with a ×63/1.4 oil immersion objective lens. Briefly prehybridisation was followed by overnight hybridization at 66 °C in a humid atmosphere. A single wash at room temperature with 2 × SSC/0.1 % Tween-20 preceded three washes at 65 °C with 0.1 × SSC. Slides were then mounted in DAPI Vectashield or processed further for dual staining of RNA and protein.

### RNA-binding UV-crosslinking assays

RNA-binding assays were carried out as described previously [12, 13]. Recombinant proteins were expressed and purified from *E.coli* (Supplementary Table 1). Magoh, SRSF2 9-101, ALYREF, hnRNP A1-like2, hnRNP K, and hnRNP F were expressed in *E. coli* and purified by Ion Metal Affinity Chromatography in 1 M NaCl containing buffers to remove potentially bound RNA from E. coli. hnRNP K was further purified by ion exchange chromatography using a Mono Q column (GE healthcare).

(GGCCCC)_5_ and (CCCCGG)_5_ RNAs were 5′ end labelled with [ɣ32P]-ATP using T4 polynucleotide kinase (Fermentas). Reaction mixes were made up in RNA binding buffer [15 mM HEPES pH 7.5, 500 mM NaCl, 5 mM MgCl2, 10 % (v/v) glycerol, 0.05 % (v/v) Tween-20] with 50 ng radiolabelled RNA and 2 µg purified recombinant protein. Mixes were incubated for 10 min at room temperature before being UV irradiated on ice at full power. Complexes were analysed by SDS/PAGE and stained with Coomassie blue before being vacuum dried and exposed on a phosphoimage screen.

### Immunohistochemistry

The following antibodies were used for IHC anti-TDP-43 (Proteintech 10782-2-AP), anti-hnRNP H/F (Abcam ab10689), anti-hnRNP A1 (Abcam ab5832, 9H10 clone), anti-SRSF2 (Abcam ab11826), anti-ALYREF (Sigma, clone 11G5), anti-nucleolin (Proteintech 10556-1-AP), and anti-hnRNP K (Abcam ab52600). Poly-GA was detected with anti-GA antibodies (mouse, clone 5F2) as previously described [18]. Poly-GR, poly-PA, poly-PR, and poly-PG were detected with antibodies provided by Stuart Pickering-Brown (Proteintech, Manchester, UK). For anti-hnRNP A1 and anti-SRSF2, antigen retrieval was performed by microwaving for 10–30 min in EDTA at pH 8.0. For all other antibodies, antigen retrieval involved 10–20 min microwave in trisodium citrate at pH 6.5 except for anti-hnRNP H/F where no specific antigen retrieval was performed. After incubation with the primary antibodies overnight at 4 °C in DEPC-treated PBS/5 % BSA slides were washed in DEPC PBS and incubated in fluorescent species-specific secondary antibodies. When dual staining of protein and RNA was performed, RNA FISH was performed first after which slides were immediately transferred to PBS/5 % BSA for protein staining.

## Results

### Relative distribution of sense and antisense RNA foci

**Fig. 1:**
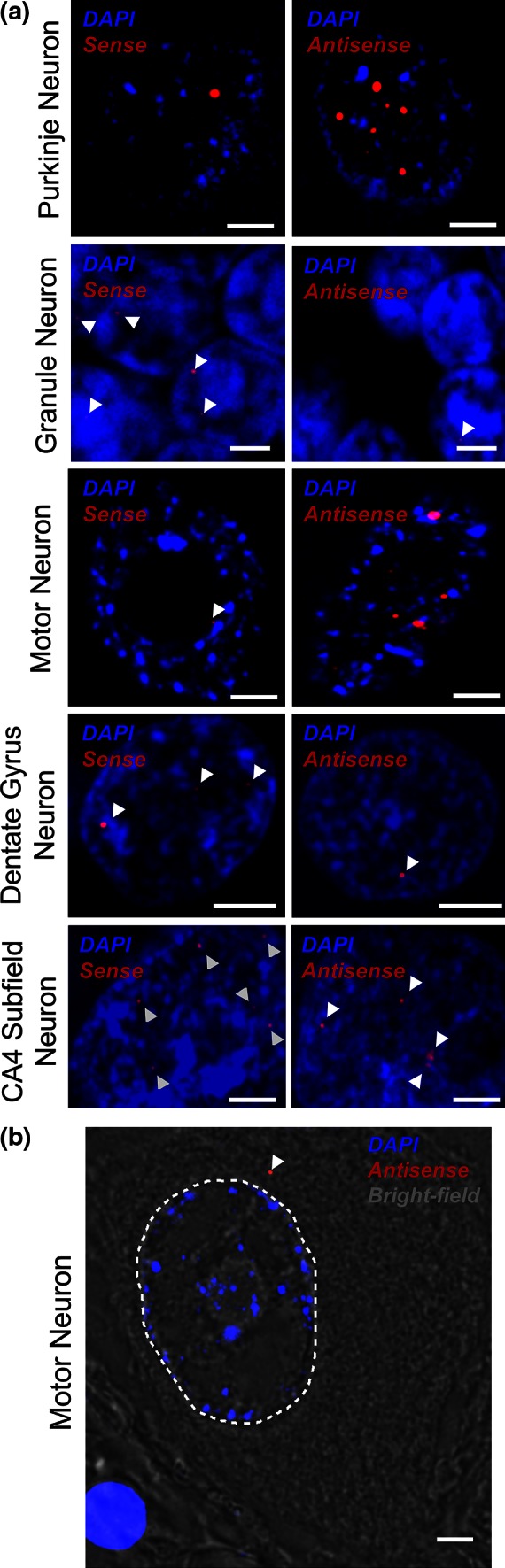
RNA FISH reveals the distribution of sense and antisense RNA foci in five neuronal populations. Representative images show that antisense RNA foci are more numerous in cerebellar Purkinje neurons and motor neurons; in contrast sense, RNA foci are more numerous in cerebellar granule neurons; neither population is more abundant in dentate gyrus neurons and CA4 subfield neurons of the hippocampus (**a**). Smaller foci are highlighted by *arrowheads*. As has been previously demonstrated for sense foci, antisense foci are occasionally present in the cytoplasm of mature motor neurons (**b**, *arrowhead*, the nuclear border is indicated by a *dotted line*). *Scale* *bar* 3 µm

The frequency of sense and antisense RNA foci was determined in five neuronal populations: Purkinje and granule neurons in the cerebellum, motor neurons of the spinal cord ventral horn, and neurons of the hippocampal dentate gyrus and CA4 subfield. These neuronal populations were chosen as they all exhibit neurodegeneration in *C9ORF72*-ALS and are characteristic of both motor (motor neurons) and extra-motor (cerebellum and hippocampus) pathology [4]. Sequential sections of tissue from *C9ORF72*-ALS cases, non-*C9ORF72* ALS cases, and controls were examined for RNA foci in a blinded manner. No RNA foci were observed in tissue from controls and non-*C9ORF72* ALS cases. Forty Purkinje neurons, forty motor neurons, >200 granule neurons, >150 dentate gyrus neurons, and >100 CA4 subfield neurons were evaluated from four C9ORF72+ ALS and/or FTD cases. The average frequency of sense and antisense foci per cell is shown in Table 2 (raw data are shown in Supplementary Table 2). Comparison between cases showed that the frequency of sense and antisense RNA foci was positively correlated in all neuronal populations i.e., cases with more sense foci per cell also had more antisense foci per cell. The exception to this was the dentate gyrus neurons where case-to-case variability was smallest (Pearson correlation coefficient: cerebellar Purkinje neurons 0.99, cerebellar granule neurons 0.6, motor neurons 0.2, CA4 subfield neurons 0.65) (Table 2). In the cerebellar populations and motor neurons but not hippocampal neurons, there was a difference between the frequency of antisense and sense foci which was consistent between cases (representative images are shown in Fig. 1a). To determine whether this difference was statistically significant, the foci count was modelled as a Poisson distribution and performing a likelihood-ratio test revealed that, within each individual case, the frequency of antisense compared to sense RNA foci was significantly higher in Purkinje neurons (likelihood-ratio test *p* < 0.05) and motor neurons (likelihood-ratio test *p* < 0.05), but significantly lower in cerebellar granule neurons (likelihood-ratio test *p* < 0.05). The fact that sense and antisense foci were relatively more abundant in different neuronal populations is against an artefact caused by differences in affinity of RNA FISH probes.

**Table 2:** Mean and standard deviation (SD) of number of sense and antisense RNA foci per nucleus in Purkinje neurons, granule neurons, motor neurons, dentate gyrus neurons, and CA4 subfield neurons in four *C9ORF72*-ALS patients

Case	Antisense (mean)	Antisense (SD)	Sense (mean)	Sense (SD)	*p* value
Purkinje neurons					
1	26.40	20.3	6.40	17.4	2.37E−14
2	4.30	4.19	1.10	2.18	0.002
3	4.60	4.50	1.30	1.95	0.002
4	6.30	5.54	1.40	1.51	6.88E−05
Granule neurons					
1	0.00	0.00	0.56	1.20	1.37E−12
2	0.03	0.17	1.10	1.41	9.63E−18
3	0.01	0.10	0.34	0.93	1.17E−07
4	0.02	0.14	0.40	0.82	3.1E−07
Motor neurons					
1	14.90	24.5	1.50	1.65	5.65E−14
2	3.00	4.06	1.00	1.25	0.02
3	3.33	3.78	1.00	0.76	0.02
4	5.40	6.52	2.44	3.40	0.02

Case	Antisense (mean)	Antisense (SD)	Sense (mean)	Sense (SD)
Dentate gyrus neurons				
1	0.65	1.90	0.88	2.34
6	1.65	3.35	0.95	1.81
7	0.89	2.42	1.39	2.23
8	1.63	5.34	0.91	1.76
CA4 subfield neurons				
1	10.3	14.7	6.55	8.68
6	3.17	6.51	1.33	1.67
7	0.50	0.97	2.72	4.90
8	6.31	10.8	9.82	13.8

In each case, antisense RNA foci are significantly more numerous in Purkinje neurons and motor neurons (likelihood-ratio test *p* < 0.05) but significantly less numerous in granule neurons (likelihood-ratio test *p* < 0.05)

As reported for sense RNA foci [3], we observed cytoplasmic antisense RNA foci even in post-mitotic mature cells such as motor neurons (Fig. 1b).

### Relative distribution of DPRs derived from sense and antisense RNA sequences

**Fig. 2:**
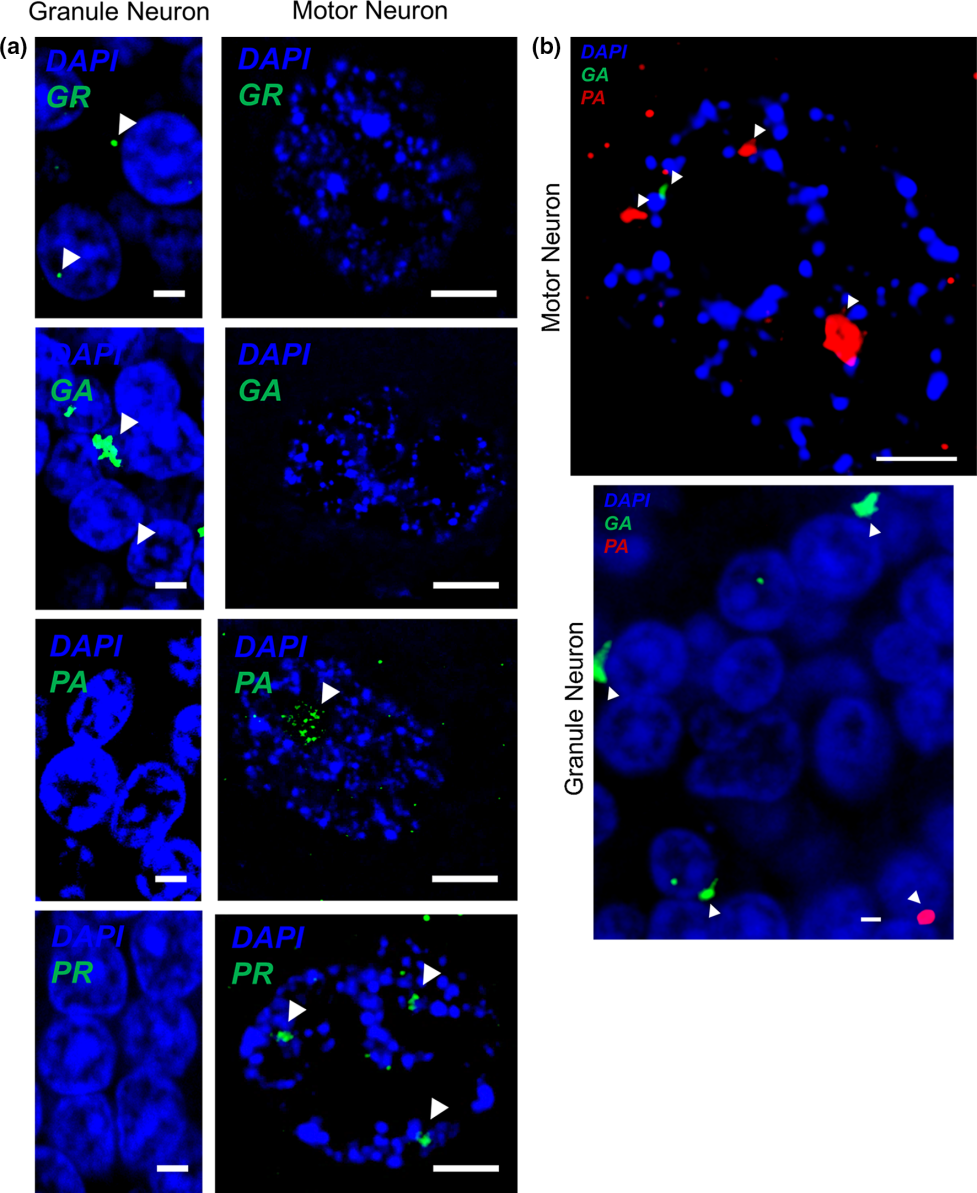
Immunohistochemistry reveals the distribution of dipeptide repeat protein containing inclusions consisting of species derived from sense and antisense repeat RNAs in two neuronal populations. Representative images showing that poly-GA and poly-GR containing inclusions are more numerous in cerebellar granule neurons, whereas poly-PA and poly-PR containing inclusions are more numerous in motor neurons. Staining was carried out individually for each protein (**a**) and then poly-GA and poly-PA were examined by dual staining (**b**). Inclusions are highlighted by *arrowheads*. *Scale bar* 3 µm

Staining of poly-GA, poly-GR, poly-PA, poly-PR, and poly-PG protein was studied in cerebellar granule neurons and motor neurons from three *C9ORF72*-ALS cases. More than 1000 granule neurons and approximately 50 motor neurons were examined in a blinded experiment. Neuronal inclusions containing sense RNA derived DPRs were only observed in granule neurons, whereas inclusions containing antisense RNA derived DPRs were only observed in motor neurons (Fig. 2a). Above background staining for poly-PG was not observed in any cells. In motor neurons, inclusions were predominantly nuclear, but in granule neurons inclusions were predominantly cytoplasmic (Fig. 2a). Background staining was examined in control and non-*C9ORF72*-ALS cases.

As a further validation, and to extend, the conclusions of this study to a larger number of cases, dual staining of poly-GA, and poly-PA protein were examined in a further blinded experiment. Approximately 1000 granule neurons and 50 motor neurons were studied from six C9ORF72+ ALS and/or FTD cases including three cases not utilized in the earlier analysis. In each case, the correct protein was determined based on the frequency of observed inclusions (Fig. 2b). Modelling the number of neuronal inclusions as a Poisson distribution and performing a likelihood-ratio test revealed that the frequency of poly-GA inclusions was significantly higher in granule neurons (likelihood-ratio test *p* < 0.01) and the frequency of poly-PA inclusions was significantly higher in motor neurons (likelihood-ratio test *p* < 0.01). The average frequencies of inclusions containing poly-GA and poly-PA protein are shown in Table 3 (raw data are shown in Supplementary Table 3).

**Table 3:** Mean and standard deviation (SD) of number of inclusions per cell containing poly-GA and poly-PA protein, in granule neurons and motor neurons from six patients with C9ORF72-disease

Case	Poly-PA (mean)	Poly-PA (SD)	Poly-GA (mean)	Poly-GA (SD)	*p* value
Motor neurons					
1	2.1	2.32	0	0	0
2	2.4	3.58	0.5	0.76	1.57E−34
4	2	1.83	0.3	0.35	0.0046
6	1.2	0.75	0	0	1.55E−21
7	1.7	1.56	0.2	0.40	0
8	4.4	2.07	0.2	0.44	0
Granule neurons					
1	0.04	0.18	0.2	0.36	2.42E−44
2	0.01	0.07	0.21	0.37	0
4	0.04	0.2	0.2	0.42	0
6	0.01	0.09	0.1	0.36	0
7	0.01	0.08	0.1	0.29	7.58E−49
8	0.01	0.12	0.1	0.34	0

In each case, poly-GA containing inclusions are significantly more numerous in granule neurons (likelihood-ratio test *p* < 0.01) and poly-PA containing inclusions are significantly more numerous in motor neurons (likelihood-ratio test *p* < 0.01)

In our previous study [3] we showed that, at a cellular level, there was no significant correlation between the presence of sense RNA foci and the presence of sense RNA-derived poly-GA inclusions. In this study, we examined the relationship between the presence of antisense RNA foci and poly-PA inclusions in fifteen motor neurons from four *C9ORF72*-ALS cases (Supplementary Table 4). As for the sense species, there was no significant correlation between the two observations (*χ*^2^, *p* = 0.83).

### Cellular distribution of RNA foci and RRM-containing proteins

We used confocal microscopy to validate in vivo some of the RRM-containing proteins that were found to interact with (CCCCGG)_4_ repeat RNA [11]. We and others have previously demonstrated colocalisation of SRSF2, hnRNP A1, hnRNP H/F, and ALYREF with sense RNA foci [3, 17]. We set out to investigate the cellular distribution of the same proteins with respect to antisense RNA foci, and we also examined nucleolin and hnRNP K which are proposed to be specific binding partners of sense and antisense foci, respectively [11].

**Fig. 3:**
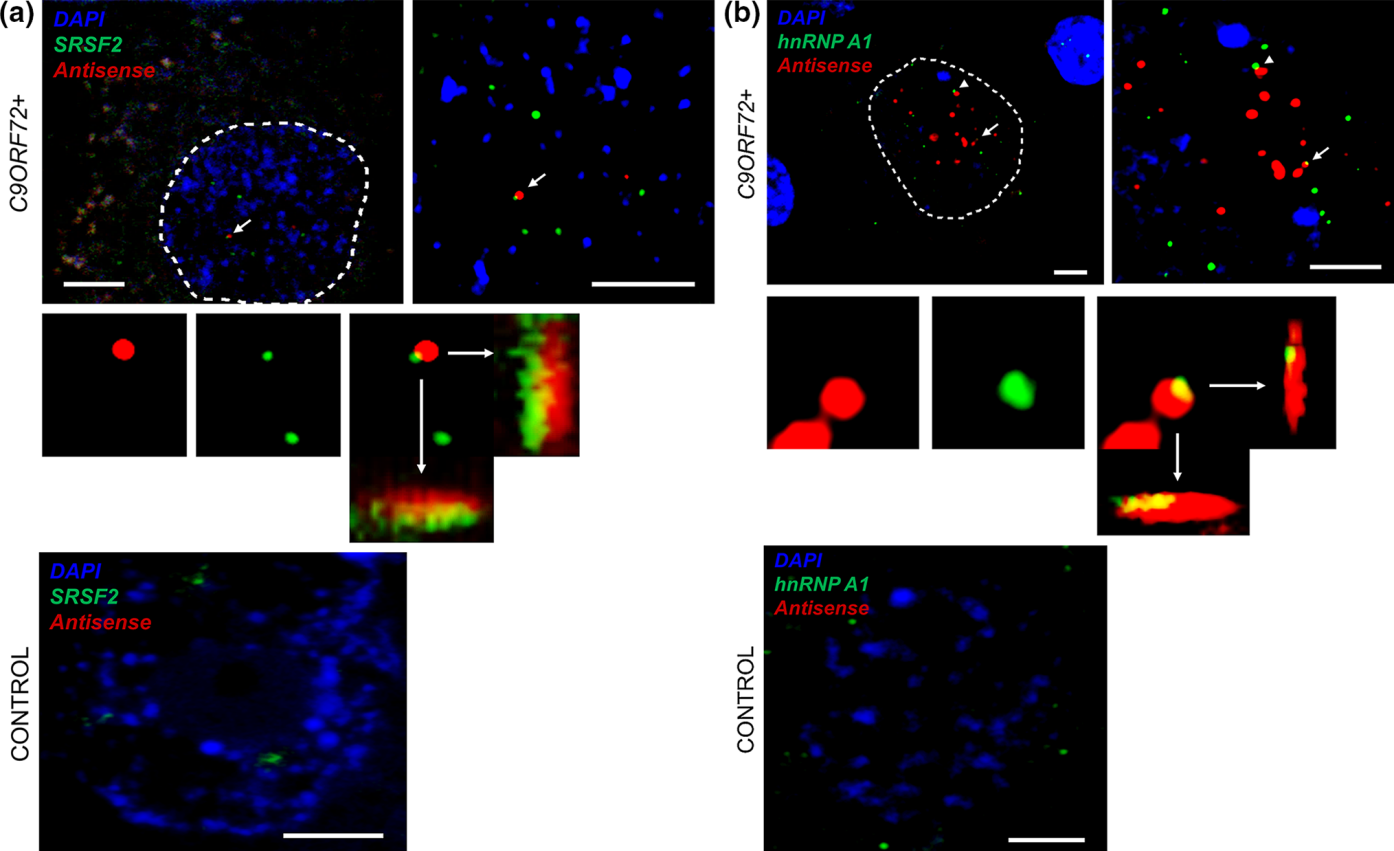

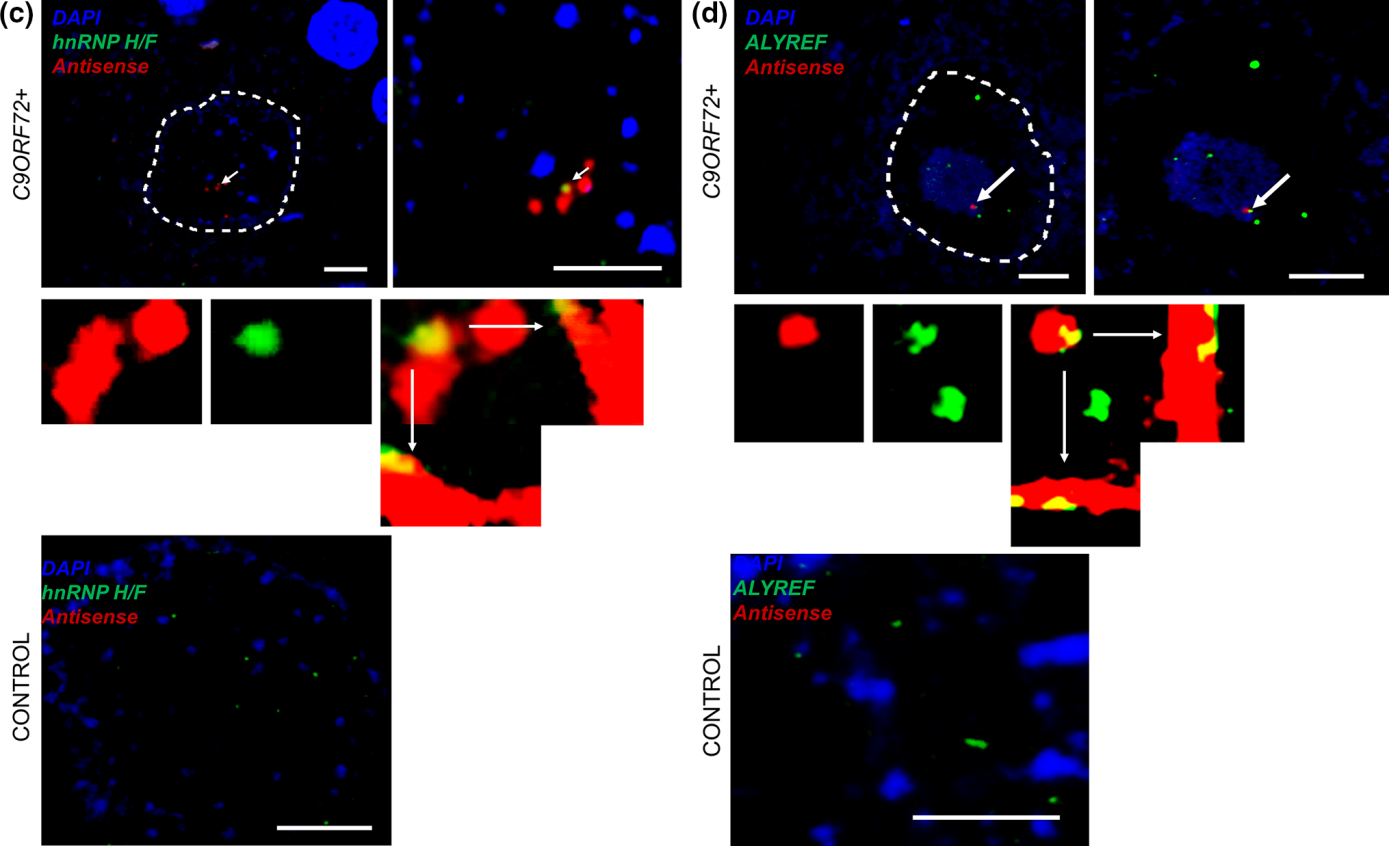

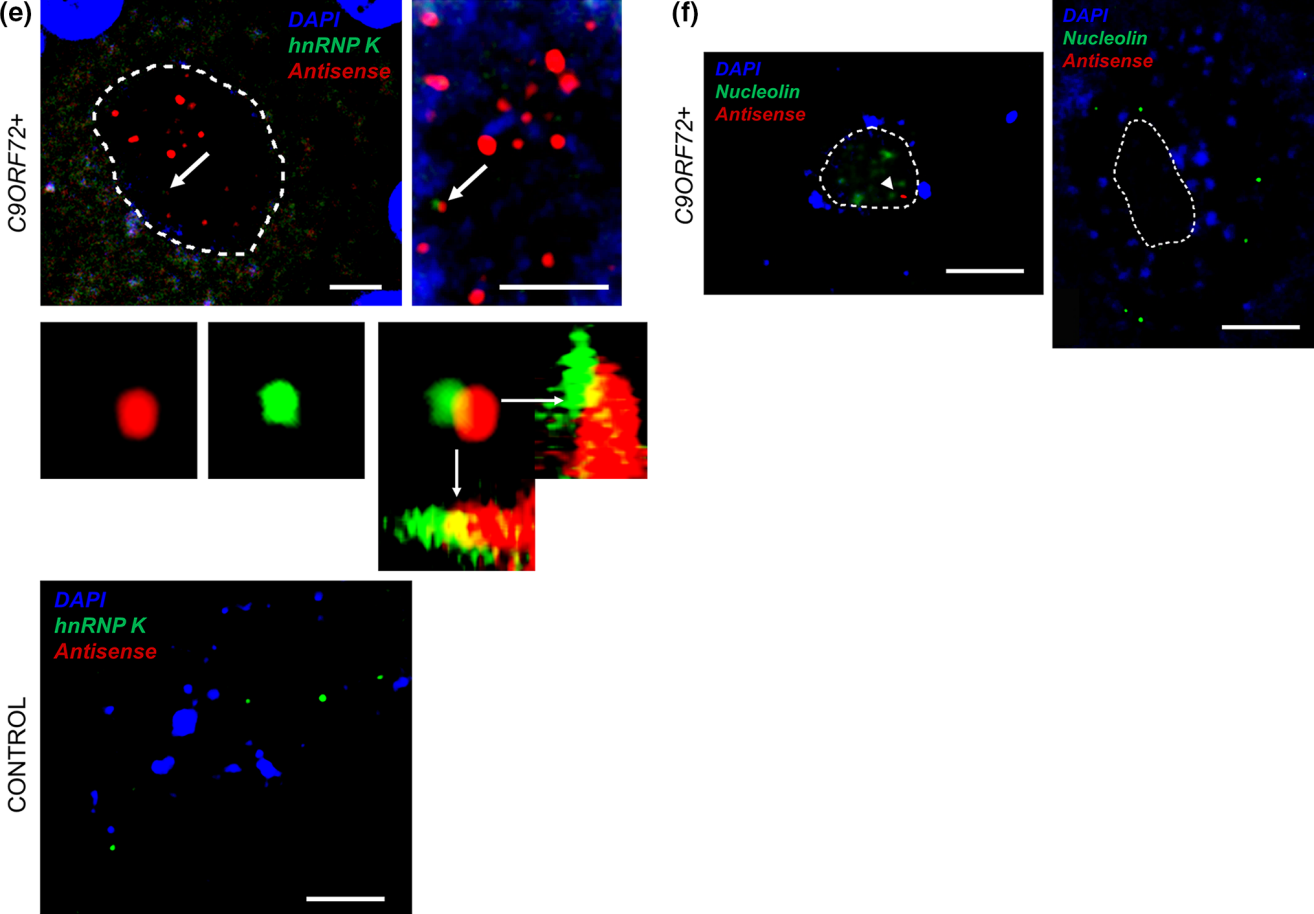
Combined RNA FISH and IHC demonstrate colocalisation of nucleolin and nuclear speckle components with antisense RNA foci in Purkinje neurons from *C9ORF72*-ALS patients and the distribution of these proteins in Purkinje neurons from control individuals. SRSF2 (**a**), hnRNP A1 (**b**), hnRNP H/F (**c**), ALYREF (**d**), and hnRNP K (**e**) are observed to colocalise with antisense RNA foci (*arrows*) in Purkinje neurons from *C9ORF72*-ALS patients. A large scale view is shown to the *left* of a zoomed-in image. Colocalisation events are enlarged including orthogonal views, and unmerged protein and RNA foci are shown for comparison. There was not a significant difference between the staining of these proteins in controls and C9ORF72+ individuals, but no antisense RNA foci are observed in controls. Nucleolin was not observed to colocalise with antisense RNA foci (**f**); moreover, the distribution of nucleolin was variable in *C9ORF72*+ Purkinje neurons. In some cells, nucleolin was prominently nucleolar (**f**, *left panel*) and in other cells it was dispersed throughout the nucleus (**f**, *right panel*, RNA focus is indicated by an *arrowhead*). The *dotted line* illustrates the nuclear border in images **a**–**e** and the nucleolar border in image **f**. Scale bar 3 µm

Approximately 50 cerebellar Purkinje neurons were examined in a blinded experiment, from a minimum of three *C9ORF72*-ALS cases. Simultaneous co-staining was carried out in parallel in non-*C9ORF72* ALS cases and neurologically normal controls. For ALYREF, hnRNP A1, SRSF2, hnRNP H/F, and hnRNP K, the overall cellular distribution was not specifically altered in *C9ORF72*+ cases except for nuclear areas where colocalisation was demonstrated (Fig. 3a–e). Haeusler et al. [11] observed disruption of nucleolin expression from the nucleolus in cell models expressing expanded *C9ORF72,* but reported a variable distribution or nucleolin in *C9ORF72*+ CNS tissue. In agreement with this, we identified *C9ORF72*+ neurons which did and did not demonstrate an altered distribution pattern of nucleolin (Fig. 3f).

**Fig. 4:**
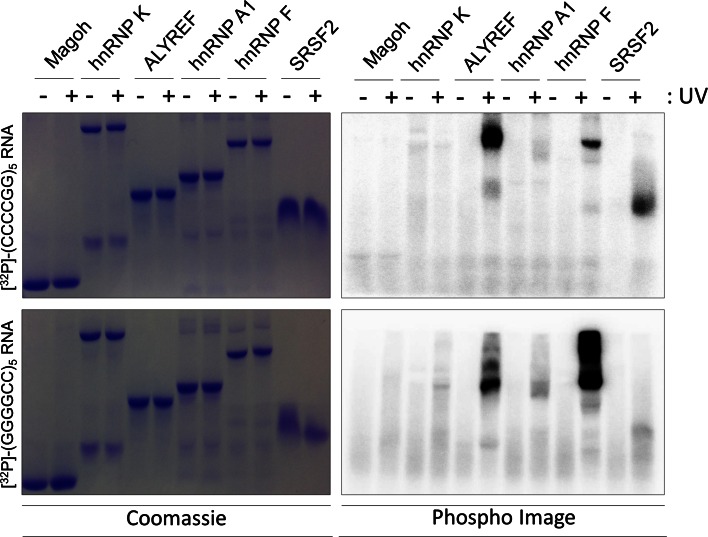
Specific and direct interactions between (GGGGCC)_5_ and/or (CCCCGG)_5_ and hnRNP A1, hnRNP F, SFRS2, and ALYREF but not hnRNP K or Magoh (negative control). Magoh, SRSF2 9-101, ALYREF, hnRNP A1-like2, hnRNP K, and hnRNP F were expressed in *E. coli* and purified (see Supplementary Table 4). (GGGGCC)_5_ (sense) and (CCCCGG)_5_ (antisense) RNA oligonucleotides were end labelled with polynucleotide kinase using [ɣ-32P]-ATP, prior to incubation with purified proteins. RNA was covalently bound (+) or not (−) following UV irradiation. The absence of radioactive signal (*right panel*, PhosphoImage) in the absence of UV irradiation demonstrates specificity of direct binding observed after UV treatment. All gels shown in the *different panels* were exposed simultaneously for the same amount of time (4 h). Note that a high molecular weight band is also observed for ALYREF due to oligomerisation properties [10]

By IHC, we demonstrated colocalisation of SRSF2, hnRNP A1, hnRNP H/F, ALYREF, and hnRNP K in cerebellar Purkinje neurons with 34, 21, 3.4, 7.8, and 8.1 % of antisense RNA foci, respectively (Fig. 3a–e). In contrast, nucleolin was not observed to colocalise with antisense RNA foci (Fig. 3f). To validate the IHC findings, we performed in vitro Ultra-Violet (UV) crosslinking assays using radiolabelled synthetic (GGGGCC)_5_ or (CCCCGG)_5_ RNA oligonucleotides, and purified recombinant proteins synthesized in *E.coli*. Unlike IHC, this allows determination of direct and specific RNA:protein interactions via the formation of covalent bonds under UV light exposure. Both sense and antisense repeat RNA were observed to directly interact with hnRNP F, hnRNP A1, ALYREF, and SRSF2 proteins although the RNA-binding activity was not equal in all cases and for hnRNP A1 was relatively low (Fig. 4). In contrast, we failed to detect any direct interactions between sense or antisense repeat RNA and hnRNP K, suggesting that the previously observed colocalisation of hnRNP K with antisense RNA foci is not due to direct binding between hnRNP K and CCCCGG-repeats. The smeared appearance of certain of the proteins on the phospho image (Fig. 4) is likely to be due to the formation of covalently bonded oligomeric protein:RNA complexes. Multiple molecules of RRM-containing proteins bind to RNA oligonucleotides via inter and intra molecular interactions. We have demonstrated a similar effect previously [10].

### Cellular distribution of RNA foci and TDP-43

**Fig. 5:**
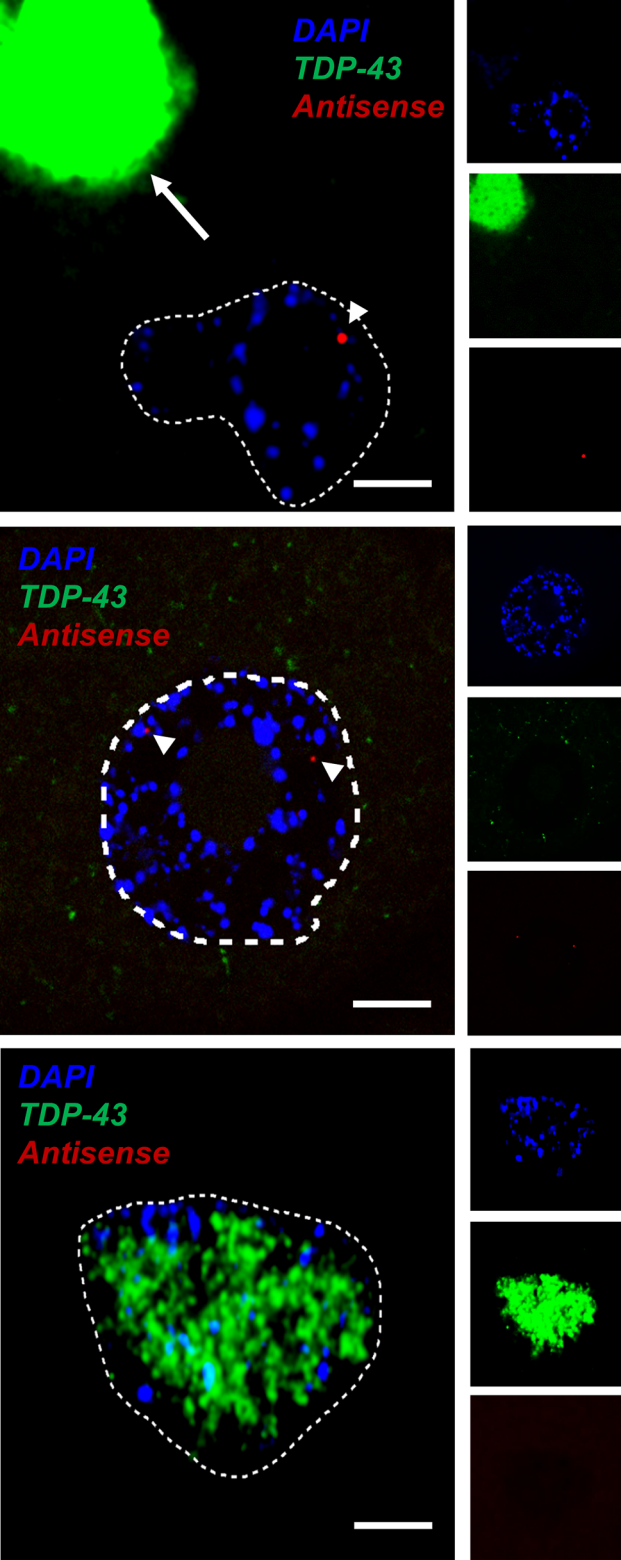
TDP-43 IHC and RNA FISH demonstrate that antisense RNA foci are significantly associated with nuclear clearance of TDP-43 in motor neurons. Representative images showing that antisense RNA foci (*arrowheads*) are significantly associated with nuclear clearance of TDP-43 in motor neurons of *C9ORF72*-ALS patients; split channel images are provided for comparison. Cleared TDP-43 may be present within a cytoplasmic inclusion (*upper panels*; RNA focus is indicated by the *arrowhead*, a compact inclusion is *arrowed*) or simply present in the cytoplasm (*middle panels*; RNA foci are indicated by *arrowheads*). In contrast, the absence of antisense RNA foci is significantly associated with the presence of nuclear TDP-43 (*lower panels*). *Scale bar* 3 µm

We also examined the association of RNA foci with depletion of TDP-43 from the nuclei of motor neurons of seven patients with *C9ORF72*-ALS. Nuclear depletion and cytoplasmic mislocalisation of TDP-43 form the pathological hallmark of most subtypes of ALS, including *C9ORF72*-mediated disease [25]. We have previously shown that the proportion of sense RNA foci+ motor neurons with and without nuclear TDP-43 is approximately equivalent (*χ*^2^, *p* = 0.75) [3]. As a direct comparison with this study, approximately fifty motor neurons were examined in FFPE sections from seven *C9ORF72*-ALS cases (Supplementary Table 5). Unlike sense RNA foci, the presence of antisense foci was significantly associated with nuclear loss of TDP-43. Seventy-seven percent of antisense foci+ motor neurons displayed loss of nuclear TDP-43 compared to 13 % of motor neurons without observable antisense foci (*χ*^2^, *p* < 0.00001) (e.g. Fig. 5). A similar experiment in hippocampal CA4 subfield neurons did not reveal a significant correlation between the presence of antisense foci and nuclear loss of TDP-43, indeed no CA4 subfield neurons exhibited complete nuclear clearance of TDP-43 (data not shown).

## Discussion

The precise mechanisms of neuronal injury in *C9ORF72*-disease appear complex, and are likely to involve RNA gain-of-function toxicity mediated by sense and antisense transcription of the GGGGCC repeat expansion (reviewed in [2, 4]). The small number of cases examined in this study and the wide variability in the phenotype of *C9ORF72*-related disease prohibits informative comparison between foci distribution and clinical phenotype, but a useful proxy is the pathological hallmark of ALS neurodegeneration: nuclear loss of TDP-43 [25]. We have demonstrated that antisense but not sense foci are significantly associated with nuclear loss of TDP-43 in motor neurons. This intriguing observation suggests that antisense RNA foci may occupy a key position in the cascade of disease pathogenesis. Moreover, examining the differences and similarities between the two species of RNA foci may shed light on important mechanisms leading to neurodegeneration.

We have demonstrated colocalisation of antisense RNA foci with SRSF2, hnRNP A1, hnRNP H/F, ALYREF, and hnRNP K, but not nucleolin. This is consistent with the work of Haeusler et al. [11]. UV-crosslinking studies confirmed that each of these interactions is direct and specific, with the exception of hnRNP K. Conflicting results between the two methodologies may arise because IHC is unable to distinguish between direct and indirect interaction. There is significant potential for indirect binding: many RRM proteins co-exist and interact within nuclear speckles. Notably of the proteins we have examined, SRSF2 colocalisation with both sense [3] and antisense foci was observed with the highest frequency, and this protein is the core component of nuclear speckles [30].

Both the IHC and the UV-crosslinking studies in this report suggest that the binding partners of sense and antisense RNA foci are not significantly different. This is also reported by others [11]. Many of these identified binding partners are localised, with SRSF2, to nuclear speckles, nuclear domains implicated in the storage, and supply of splicing factors to active transcription sites [30]. Neuromuscular diseases, including type 1 myotonic dystrophy (DM1), have been associated with depletion of normal components of nuclear speckles [1, 29]. Sequestration of these proteins by sense or antisense RNA foci and consequent disruption of the normal function of these essential nuclear organelles might be a key event in the pathophysiology of *C9ORF72*-mediated neurodegeneration. If so, our results would predict that both species of RNA foci should be equally toxic. This is consistent with observed toxicity of sense foci in various model systems [8, 16, 17, 21, 28]. This led us to ask whether the key difference might not be in the interactions of the foci themselves, but in the neuronal populations in which sense and antisense foci are expressed.

In all cases, the relative frequency of sense and antisense foci varied consistently and significantly between neuronal populations. Importantly in motor neurons, the primary target of pathology in ALS, antisense foci are more abundant than sense foci. Therefore, we suggest that the key event determining toxicity leading to TDP-43 mislocalisation, of antisense as opposed to sense RNA foci, might be a propensity to produce antisense foci mediated by cell-specific transcriptional regulation. Alternatively sense RNA foci might be degraded at a higher rate than antisense RNA foci. *In*-*vitro* studies have suggested that both sense and antisense RNA sequences form complex secondary structures including G-quadruplexes and hairpin loops [11]. These secondary structures may help stabilize the RNA foci and prevent degradation.

It is interesting that two populations of relatively large neurons, motor neurons and cerebellar Purkinje neurons, exhibited antisense RNA foci at a higher frequency than sense RNA foci, in contrast to the smaller cerebellar granule neurons. This suggests that our observations may be related to some property correlated with neuronal size. However, in the hippocampus, neither the larger CA4 subfield neurons nor the smaller dentate granule neurons exhibited either species of RNA foci at a consistently higher frequency.

We observed antisense foci in the cytoplasm of motor neurons, which is consistent with aberrant nuclear export and may be a key step in the facilitation of proposed repeat associated non-ATG translation to produce DPR species [24]. We made a similar observation with respect to sense foci [3] and we suggest that interaction between repeat RNA and mRNA export adaptors, such as ALYREF, might override the normal nuclear retention of pre-mRNA species. Recent studies consistent with a key role for DPRs in the pathogenesis of *C9ORF72*-mediated neurodegeneration [15, 20, 22] suggest that this represents an attractive therapeutic target.

We have demonstrated that the frequency of sense and antisense foci is usually correlated i.e. a patient with more sense foci will also have more antisense foci. This is particularly interesting in case 1 from our analysis (Table 2) who displayed a relatively high frequency of sense and antisense RNA foci in the cerebellum and CA4 subfield neurons of the hippocampus, which are both extra-motor areas. Case 8 also exhibited a relatively high frequency of sense and antisense RNA foci in CA4 subfield neurons; the frequency of RNA foci in the cerebellum of case 8 was not quantified. In contrast to the other cases examined these patients displayed extra-motor disease clinically as well as pathologically: clinical FTD was diagnosed with (case 1) and without (case 8) ALS (Table 1). This is consistent with a correlation between the development of RNA foci in specific neuronal subtypes and clinical presentation, but this hypothesis will require validation in a larger number of FTD and ALS cases.

Finally, varying frequency of the expression of sense and antisense repeat RNA has implications for the formation of specific DPRs. Our observations of all five DPRs are consistent with our conclusions relating to the expression of sense and antisense RNA foci. In cerebellar granule neurons, where sense RNA foci are more abundant, there is a higher frequency of sense-RNA derived DPR inclusions; and in motor neurons where antisense RNA foci are more abundant, there is a higher frequency of antisense RNA-derived DPR inclusions. Therefore, we suggest that, at least in these neuronal populations, translation of the sense and antisense derived proteins occurs in different quantities depending of the relative availability of RNA repeat molecules. The results of the present study potentially explain the observations of others that inclusions containing poly-GA protein are much more abundant in certain neuronal populations including cerebellar granule neurons [18]. However, Davidson et al. [6] failed to demonstrate antisense RNA derived DPR inclusions in Purkinje neurons of the cerebellum and dentate gyrus neurons of the hippocampus. This contrasts with our demonstration of antisense RNA foci in both of these populations, particularly in the cerebellar Purkinje neurons which we found to show a preference for exhibiting antisense rather than sense RNA foci. This variation between neuronal populations might be explained by variability in control of nuclear export of repeat RNA species; in this context, it is interesting that mutations in *hGle1*, a mRNA export adaptor, have recently been shown to cause selective death of motor neurons [14].

In our previous study we showed that there was no significant correlation between the presence or absence of nuclear sense RNA foci in cerebellar granule neurons and whether or not those cells contain a cytoplasmic inclusion positive for poly-GA [3]. Similarly in this study we have shown that there is no significant correlation between the presence or absence of nuclear antisense RNA foci in motor neurons, and whether or not those cells contain an inclusion positive for poly-PA. This suggests that our population level conclusion in this study, that neuronal populations have a propensity to produce either sense or antisense RNA derived foci and DPRs, does not apply at a cellular level. Thus, whilst both RNA foci and DPRs are derived from the same RNA molecules, the processes by which this RNA is stabilised into a focus or exported for translation are probably different or even mutually exclusive—indeed work from Gendron et al. [9] suggested that this might be the case. So if motor neurons have a preference for antisense transcription of the *C9ORF72* expansion then the motor neuron population will express higher levels of antisense RNA derived foci and DPRs, but not necessarily within the same individual cells.

Our work highlights that any therapeutic approach to *C9ORF72*-ALS should consider the presence of antisense RNA foci in motor neurons. An antisense oligonucleotide approach has been proposed as a therapeutic option in *C9ORF72*-disease [8, 28]. We suggest that both sense and antisense RNAs should be targeted, as has been proposed by others [16]. Indeed, in relation to the relative selective vulnerability of motor neurons in *C9ORF72*-mediated pathology in vivo, targeting the antisense foci may be even more important than targeting sense foci. A limitation of our study is the reliance on post-mortem tissue which represents end-stage disease and may exclude the most vulnerable cells which have already been lost; as such we await validation of our findings in model systems.

## Electronic supplementary material

Supplementary material 1 (PDF 103 kb) Supplementary Table 1: Raw counts of the presence/absence of nuclear antisense RNA foci relative to TDP-43 distribution in motor neurons from *C9ORF72*-ALS cases by TDP-43 IHC and RNA FISH

Supplementary material 2 (XLS 58 kb) Supplementary Table 2: Raw counts of sense and antisense nuclear RNA foci in motor neurons, cerebellar granule and Purkinje neurons, hippocampal dentate gyrus neurons and CA4 subfield neurons, from *C9ORF72*+ cases

Supplementary material 3 (XLS 29 kb) Supplementary Table 3: Raw counts of poly-GA and poly-PA inclusions in motor neurons and cerebellar granule neurons from *C9ORF72*+ cases

Supplementary material 4 (XLS 24 kb) Supplementary Table 4: Plasmids used in this study

Supplementary material 5 (XLS 28 kb)
