# Supplementary material for: Antisense RNA foci in the motor neurons of C9ORF72-ALS patients are associated with TDP-43 proteinopathy
Source: Acta Neuropathol. 2015 May 6;130(1):63–75. doi: 10.1007/s00401-015-1429-9 (PMC4468790; doi:10.1007/s00401-015-1429-9)
Supplement: Supplementary file 1 — Supplementary material 1 (PDF 103 kb) Supplementary Table 1: Raw counts of the presence/absence of nuclear antisense RNA foci relative to TDP-43 distribution in motor neurons from C9ORF72-ALS cases by TDP-43 IHC and RNA FISH [file 401_2015_1429_MOESM1_ESM.pdf]

**Plasmid name**	**Description**	**Tag(s)**	**Source**
pET24b-Magoh	Full length *Magoh* cloned as NdeI/XhoI PCR fragment into pET24b	6His (3')	[5]
pET24b-SRSF2/SC35 (9-101)	*SC35 28-303* cloned as NdeI/XhoI PCR fragment into pET24b	GB1/6His (5')	[26]
pET24b-ALYREF	Full length *ALYREF* cloned as NdeI/XhoI PCR fragment into pET24b	6His (3')	[3]
pET24b-hnRNPA1-like2	Full length *hnRNPA1L2* cloned as NheI/XhoI PCR fragment into pET24b	6His (3')	This study
pET24b-hnRNPF	Full length *hnRNPF* cloned as NdeI/XhoI PCR fragment into pET24b	6His (3')	This study
pET24b-hnRNPH1	Full length *hnRNPH1* cloned as NdeI/XhoI PCR fragment into pET24b	6His (3')	This study
pET24b-hnRNPK	Full length *hnRNPK* cloned as NheI/XhoI PCR fragment into pET24b	6His (3')	This study
